# Interventional treatment for post-thrombotic chronic venous obstruction: Progress and challenges

**DOI:** 10.1016/j.jvsv.2024.101910

**Published:** 2024-05-20

**Authors:** Mohammad E. Barbati, Efthymios D. Avgerinos, Domenico Baccellieri, Suat Doganci, Michael Lichtenberg, Houman Jalaie

**Affiliations:** a Clinic of Vascular and Endovascular Surgery, RWTH Aachen University Hospital, Aachen, Germany; b Athens Medical Center, Clinic of Vascular and Endovascular Surgery, Athens, Greece; c Vascular Surgery Unit, IRCCS San Raffaele Scientific Institute, Milan, Italy; d Department of Cardiovascular Surgery, University of Health Sciences, Ankara, Turkey; e Department Angiology, Klinikum Hochsauerland, Arnsberg, Germany

**Keywords:** Chronic venous obstruction, Post-thrombotic syndrome, Venous stenting, Patient selection, Procedural techniques

## Abstract

Chronic venous obstruction, including nonthrombotic iliac vein lesions and post-thrombotic syndrome, presents a significant burden on patients' quality of life and health care systems. Venous recanalization and stenting have emerged as promising minimally invasive approaches, yet challenges in patient selection, procedural techniques, and long-term outcomes persist. This review synthesizes current knowledge on the interventional treatment of post-thrombotic syndrome, focusing on the evolution of endovascular techniques and stenting. Patient selection criteria, procedural details, and the characteristics of dedicated venous stents are discussed. Particular emphasis is given to the role of inflow and other anatomical considerations, along with postoperative management protocols for an optimal long-term outcome.

Chronic venous obstruction (CVO) refers to the obstruction of venous flow, typically caused by a partial or complete blockage of the venous system leading to reduced flow and increased pressure in the affected veins.^1^^,^^2^ CVO is a general term that encompasses various conditions, including post-thrombotic syndrome (PTS) and nonthrombotic iliac vein lesions (NIVLs). PTS is a common complication of deep vein thrombosis (DVT), and it is estimated that ≤50% of patients with iliofemoral DVT will develop it within 2 years of their initial diagnosis.^3^^,^^4^

The symptoms of PTS can vary depending on the location and extension of the obstruction. The most common symptoms of PTS include swelling, pain, claudication and skin changes such as discoloration or ulceration. These symptoms can have a significant impact on an individual's quality of life (QoL) and may require a range of treatments to manage effectively.^5^, ^6^, ^7^, ^8^, ^9^

Venous recanalization and stenting have become a promising minimally invasive treatment for patients with PTS, offering rapid symptom relief and long-term benefits compared with conservative management^10^ and traditional open surgical techniques.^11^ The advancements in endovascular technology and the use of dedicated venous stents have enabled more precise, less invasive interventions with a lower complication risk and faster recovery times.^5^^,^^12^^,^^13^ Venous stenting offers a high rate of technical success, accompanied by minimal complications during and after the procedure.^14^, ^15^, ^16^, ^17^, ^18^ Additionally, iliac stenting has been particularly effective, significantly enhancing the healing of ulcers.^19^, ^20^, ^21^ However, venous stenting also has challenges, including the need for individual patient assessment and understanding long-term outcomes in various populations. Proper patient selection, recanalization techniques, stent sizing and placement, as well as the postprocedural treatment component are critical components of the procedure.^22^, ^23^, ^24^, ^25^, ^26^

## Patient selection for invasive pts management

In the management of PTS, the appropriate selection of patients for intervention and stent deployment is crucial for successful outcomes. It is important to consider the severity and duration of symptoms. Before considering intervention, patients should have trialed conservative treatments, including compression therapy, exercise, and limb elevation.^11^ The severity of symptoms indicates the necessity for intervention to alleviate patients' suffering and prevent further complications. Patients with debilitating symptoms may be more willing to accept the potential risks of the procedure, because the benefits outweigh the adverse outcomes.^27^ Furthermore, a comprehensive evaluation of the patient's comorbidities is essential in determining the appropriateness of intervention. Factors such as age, overall health status, and coagulation disorders should be considered to ensure that the benefits of intervention outweigh the potential risks.^3^ The link between age and the onset of PTS remains ambiguous. Age as a factor in PTS presents a mixed picture. Although a number of studies have not detected any correlation between age and PTS,^28^^,^^29^ variations in the upper age limits used for study cohorts complicate direct comparisons. However, this finding is contradicted by research suggesting that older age may actually be associated with a lower risk of developing PTS.^30^^,^^31^ Older patients may have a higher risk of comorbidities, such as cardiovascular disease, diabetes, and renal dysfunction, which could influence the decision-making process for these interventions.^32^ Conversely, younger patients may have a longer life expectancy, necessitating careful consideration of the durability and long-term effects of stenting in this group. Therefore, a comprehensive evaluation of each patient's age, overall health status, and anticipated benefits and risks of venous recanalization and stent placement is crucial for optimal decision-making. The decision to test all patients with PTS for thrombophilia is a matter of debate. Negative testing for thrombophilia may falsely reassure clinicians, leading to the cessation of anticoagulation in patients at high risk for recurrence, while finding a thrombophilia in a patient at high bleeding risk may lead to continued anticoagulation, owing to an overestimation of the risk conferred by the condition.^33^ Therefore, the decision to test for thrombophilia in patients with PTS should be based on individual clinical assessment and guided by the potential impact on management, rather than routine testing for all patients. Patients with antiphospholipid syndrome, particularly those with triple positivity or a history of arterial thrombosis, may benefit from thrombophilia testing to tailor the anticoagulation therapy. Most guidelines currently recommend using warfarin over direct oral anticoagulants in patients with antiphospholipid syndrome.^34^ It is recommended for patients with unexplained venous thromboembolism at ages of <40 to 45 years, focusing on detecting common and less well-established causes.^35^^,^^36^

## Potential risks of venous intervention and stent implantation in patients with pts

Weighing the potential benefits against the risks is central to the decision-making process, particularly the risk of stent thrombosis and the patient's ability to manage post-operative anticoagulation therapy. Bleeding events, typically minor, and in-stent stenosis or occlusion are potential complications that must be balanced against the expected symptomatic relief and QoL improvements.^7^^,^^22^^,^^25^^,^^37^^,^^38^

The mechanical stress exerted on stents placed within the venous system can occasionally lead to stent fracture. Although not all fractures are symptomatic, their occurrence may result in acute venous obstruction and can compromise the long-term patency of the stented segment.^39^ Stent fractures have been suggested to be associated with closed-cell stent designs or segments, whereas open-cell or braided stents demonstrate a markedly lower incidence of such fractures.^40^ Additionally, compression by the inguinal ligament or possibly pelvic bones during leg movement have been suggested as other probable causes of stent fracture.^41^ To mitigate this risk, stents with enhanced fracture resistance and improved design have been developed, such as self-expanding nitinol stents. However, many venous stents are relatively new to the market, and long-term data on their efficacy and safety are still emerging. This lack of data can make it challenging to predict long-term outcomes and durability of the venous stents.

One of the more concerning risks is stent migration, which can occur if the stent is not appropriately sized or is not adequately secured within the vein. Although rare, stent migration can lead to severe complications or suboptimal treatment of the affected area or cause new obstructions elsewhere, necessitating further intervention.^23^ To minimize the risk of migration, careful attention should be given to proper stent sizing, selection, and placement techniques, ensuring optimal fixation within the target vein.

In some cases, venous intervention and stent implantation may lead to the development of contralateral thrombosis. This complication, although not initially recognized as a significant risk, has emerged as a notable concern with incidences ranging from 1.1% to 2.2%, and in some instances as high as 15.6%.^42^ The risk of contralateral DVT seems to be influenced by factors such as pre-operative contralateral internal iliac vein thrombosis, preexisting inferior vena cava (IVC) filters, anticoagulation noncompliance, and the extension of the stent into the IVC obstructing flow in the contralateral iliac vein—a phenomenon termed jailing.^42^, ^43^, ^44^, ^45^ The use of intravascular ultrasound (IVUS) examination can aid in an accurate assessment of the iliocaval confluence for precise stent placement and potentially reducing the risk of contralateral DVT.^46^, ^47^, ^48^

Another potential complication can be back pain after venous intervention and stent implantation. This symptom can occur owing to the stretching and compression of the venous wall, irritation of nerve fibers, or secondary to the procedure itself with no clear correlation with stent size. Adequate pain management strategies and patient education are important to alleviate discomfort and enhance patient satisfaction. The reason is possibly the distension of the vein or the pressure of the expanding stent against surrounding tissues. This pain is typically self-limiting, but may occasionally require medical management.^49^, ^50^, ^51^

The patient's preference, informed consent, and expectations regarding the outcomes, including the potential need for reintervention and lifestyle modifications after the procedure, are integral to this decision.^8^^,^^52^ This comprehensive assessment ensures that patients selected for venous stenting are those most likely to benefit from this approach, leading to improved symptomatic outcomes and health-related QoL.^10^^,^^53^, ^54^, ^55^

## Procedure overview

The primary goal of venous recanalization is to improve venous return and decrease venous hypertension. By restoring blood flow, the treatment aims to alleviate PTS symptoms^56^^,^^57^ and prevent PTS progression to its severe manifestation like nonhealing ulcer.^58^

Venous recanalization begins with an initial venography to confirm the level and location of venous obstruction. Vascular access for the procedure is typically obtained through the femoral vein (FV), popliteal vein, and/or the right internal jugular vein. After this, intravenous heparin is administered using weight-based dosing (50 units/kg) and targeting a partial thromboplastin time of 1.5 to 2.0 times the upper limit of normal. For the initial traversal of the occluded segment, the use of hydrophilic guidewires and support catheters with a strong track record of crossing tight lesions is recommended. Support catheters, particularly those with a braided or reinforced construction, provide additional pushability and stability for guidewire advancement. When conventional guidewires and catheters fail to cross the occluded segments, using special peripheral crossing set such as Cook Medical's TriForce (Cook Medical, Bloomington, IN), steerable Guiding Sheath like DESTINO (Oscor Inc., Palm Harbor, FL) can give more stability during recanalization. Additionally, sharp recanalization becomes a vital alternative. This technique involves the use of a stiff, sharp-tipped device, such as back of the 0.018 stiff guidewire or TIPS needle (W. L. Gore & Associates, Flagstaff, AZ) to puncture through the occlusion under fluoroscopic guidance. Other devices which can be used for sharp recanalization are BeBack (Bentley InnoMed GmbH, Hechingen, Germany) or Traversa (Veinway Ltd., Yehuda, Israel) with a steerable needle. It is often necessary in such cases to place a balloon catheter via an alternative access point on the other side of the occlusion ensuring accurate alignment and minimizing the risk of vessel perforation.^59^, ^60^, ^61^ After crossing the occlusion, it is crucial to confirm the right positioning of the wire inside the iliocaval system to avoid complications during venous stenting procedures. Real-time fluoroscopic control with both frontal and lateral radiographic views is necessary. The lateral view is particularly important, because it helps to avoid the risk of following collateral veins, which may inadvertently lead into the spinal canal. This can consequently lead to dangerous complications, such as intraspinal bleeding.^62^, ^63^, ^64^ Wire escalation to stiffer and more supportive wires, such as the Amplatz Super Stiff wire (Boston Scientific, Marlborough, MA), is often necessary to facilitate the subsequent interventions. Predilation plays a critical role and should be approached methodically. Starting with a smaller balloon can aid in the passage of larger balloons. This strategy can avoid the risk of rupturing bigger balloon and difficulty in retrieving it. Stenting, particularly with self-expanding stents, should be performed with precision to ensure adequate wall apposition and coverage of the entire lesion. Postdilation is necessary to optimize stent expansion and apposition against the venous wall. Both predilation and postdilation should be carried out with a balloon size matched to the nominal diameter of the stent.

Additionally, it is recommended to routinely use IVUS imaging for complimentary intraoperative assessment of the iliofemoral and caval pathology. It should be noted that IVUS imaging will not be useful to evaluate the post-thrombotic occluded vein for stent sizing. IVUS’s role in these cases is the precise evaluation of the proximal and distal extents of occlusion and accurate mapping of the landing zones.^65^ Finally, venoplasty and stenting are performed on the obstructed segments, followed by completion venography and IVUS examination to confirm unobstructed venous patency and optimal stent positioning.^66^^,^^67^

## Venous stents

Dedicated venous stents have emerged as a vital tool in the management of CVO, particularly in cases where conservative treatments have proven insufficient. The market for dedicated venous stents has expanded in recent years, leading to the development of several specialized stent options. Some of the most notable dedicated venous stents currently available include Zilver Vena (Cook Medical), Sinus Venous (Optimed, Newtown, PA), Venovo (BD, Franklin Lake, NJ), Abre (Medtronic, Minneapolis, MN), BeYond (Bentley), Blueflow (Plus Medica Dusseldorf, Germany), and Vesper DUO (Philips, Best, the Netherlands). Each of these stents has distinct properties and characteristics, catering to different clinical needs and anatomical considerations.

Reported outcomes for dedicated venous stents have generally been positive, with high rates of technical success and significant improvement in patient symptoms. For instance, the Zilver Vena stent has demonstrated primary patency rates of approximately 90% at 1 year and excellent safety profiles.^68^ The Sinus Venous stent has shown promising results in managing iliofemoral venous obstructions, with primary patency rates of up to 90% at one year.^69^ Although a randomized controlled trial (RCT) demonstrated improvement in specific QoL measures after stenting in symptomatic CVO patients at 12 months, we acknowledge the current challenge posed by the scarcity of RCTs offering level 1 evidence in this domain. The study's significance is underscored by its contribution to the body of evidence supporting stenting efficacy, which, as it stands, holds a Level B, Class IIa of evidence in practice guidelines. This finding underscores the need for further high-level RCTs to establish more definitive evidence for venous stent indications.^10^ The Venovo stent has achieved primary patency rates of approximately 88% at 1 year and 84% at 3 years with sustained QoL improvement.^37^^,^^70^, ^71^, ^72^ The 36-month final results from the ABRE clinical study showed that the effectiveness after treatment with the Abre venous stent was sustained through 36 months with an estimated primary patency rate of 81.6% and freedom from clinically driven target lesion revascularization rate of 89.3%.^73^ The BeYond and Blueflow stents have also demonstrated favorable outcomes in clinical studies, with high patency rates and improvements in patient symptoms, further expanding the range of viable stent options for physicians and patients.^74^ Table summarizes the available reported patency rates of dedicated venous stents at the pivotal trials.^23^^,^^68^^,^^73^, ^74^, ^75^, ^76^, ^77^ Despite the absence of head-to-head trials and the challenging task of comparing investigational device exemption studies, it is evident that high flexibility, high radial resistive strength, and compression resistance are vital attributes for venous stent. The Wallstent, which was originally used in an off-label capacity, featured a braided design to decrease the risk of fractures. Modern stent designs now use the natural strength of nitinol to achieve the same goal. In terms of dimensions, the Abre and Venovo stents offer the largest diameters (of ≤20 mm), albeit still insufficient for caval stenting, a niche where the Wallstent's 22 and 24 mm and sinus-XL with large diameters (from 16 to 36 mm) remain relevant for the treatment of caval obstructions. In terms of clinical outcomes, all approved venous stents have demonstrated improvements in QoL and functioning. Although fracture rates may vary slightly, they are generally low across all stents. Ultimately, the choice of venous stent often relies on various factors, including available hospital inventory and physician experience and preference.

**Table:** Overview of dedicated venous stents and their patency rates

Stent	Company name	Total No. of patients	PTS: NIVL: DVT	Patients with infrainguinal stenting	Patency rates at 12 months		
					Primary patency	Primary assisted patency	Secondary patency
Venovo Venous Stent System	BD Interventional	170	93 (54.7): 77 (45.3): -	15 (8.8)	88.6		
Zilver Vena Venous Self-Expanding Stent	Cook Medical	243	60 (24.7): 103 (42.4): 80 (32.9)	79 (23.5)	89.9	90	98.9
Abre Venous Self-Expanding Stent System	Medtronic	200	95 (47.5): 72 (36): 33 (16.5)	88 (44)	88	91.8	92.9
sinus-Obliquus	Optimed Medizinische Instrumente GmbH	60	60 (100): -: -	42 (70)	83	90.6	98.1
sinus-Venous		75	40 (53.3): 35 (46.7): -	33 (44)	92	99	100
Blueflow Venous Stent	Plus Medica GmbH	67	37 (55.2): 21 (44.8): -	24 (35.8)	79.8		

## Role of inflow

To appreciate the significance of inflow in stent outcomes, it is essential to understand the factors currently known to affect the success of stent implantation. These include stent design, material, and reconstruction technique. However, the role of blood flow in venous health has become increasingly relevant in recent years, prompting further investigation into the impact of inflow quality on the long-term results of stent implantation.^25^^,^^73^

The FV and deep FV play a vital role in inflow, returning blood from lower extremities to the stented tract.^78^ The quality of inflow in these veins can be influenced by factors such as vessel diameter, the presence of valves, the degree of obstruction, and the extension of the pathology. A clear relationship exists between inflow quality and stent outcomes, with poor inflow contributing to suboptimal results.^64^^,^^79^

Clinical evidence supports the importance of inflow quality in determining stent outcomes. Several studies have investigated the impact of inflow on treatment success, with results indicating that patients with better inflow quality tend to experience more favorable long-term outcomes after stent implantation.^37^^,^^80^, ^81^, ^82^

In the context of patient care and treatment planning, the evaluation of inflow quality before stent implantation can provide valuable information to guide clinical decision-making. Assessing inflow quality may help to identify patients who could benefit from interventions and who may not. In some patients with nonhealing ulcers, it might be necessary to optimize inflow by additional treatment tools like angioplasty of FV.^83^ By improving inflow quality, it may be possible to enhance long-term stent outcomes and decrease the risk of complications for patients with CVO.

## Anatomical considerations

The location and extent of the venous obstruction should be evaluated using imaging techniques like duplex ultrasound examination, magnetic resonance venography, or computed tomography venography. Despite the valuable role of venography and IVUS examination during interventions for their detailed luminal and adjacent structural information, they are not used as standalone preoperative diagnostic tools owing to their invasive nature. Instead, their use is reserved for the intraoperative setting, where they can guide therapeutic decisions after preoperative evaluation. This strategy ensures optimal care by using the appropriate imaging modalities at the correct junctures, thereby maximizing diagnostic efficacy and minimizing unnecessary invasive procedures.

**Fig 1:**
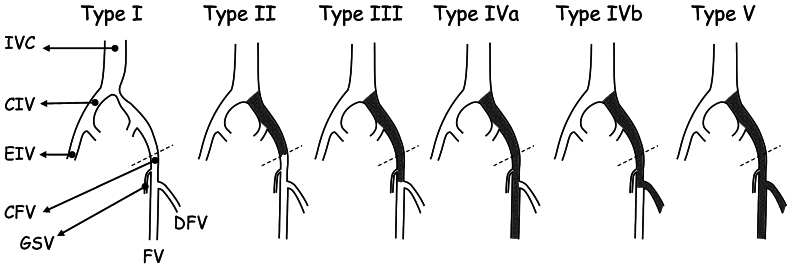
Diagram of the newly suggested categorization for chronic venous obstruction (*CVO*) along the iliofemoral pathway. The classification is divided into five types. *CFV*, common femoral vein; *CIV*, common iliac vein; *DFV*, deep femoral vein; *EIV*, external iliac vein; *FV*, femoral vein; *GSV*, great saphenous vein; *IVC*, inferior vena cava.

The anatomical extension of CVO below the inguinal ligament is correlated with stent patency in patients who undergo recanalization. Jalaie et al^84^ proposed classification system for iliofemoral CVO, which may help to predict outcomes after intervention and standardize reporting in future studies. Type 1 CVOs (NIVLs) had the highest patency rates, whereas type 5 (severe extent of pathology with involvement of FV and deep FV [DFV]) had the lowest. This classification system for iliofemoral CVO has clinical implications in predicting the outcome by estimating the quality of inflow based on distal extension of the disease and involvement of main inflow veins (Fig 1).

## Defining the proximal landing zone

**Fig 2:**
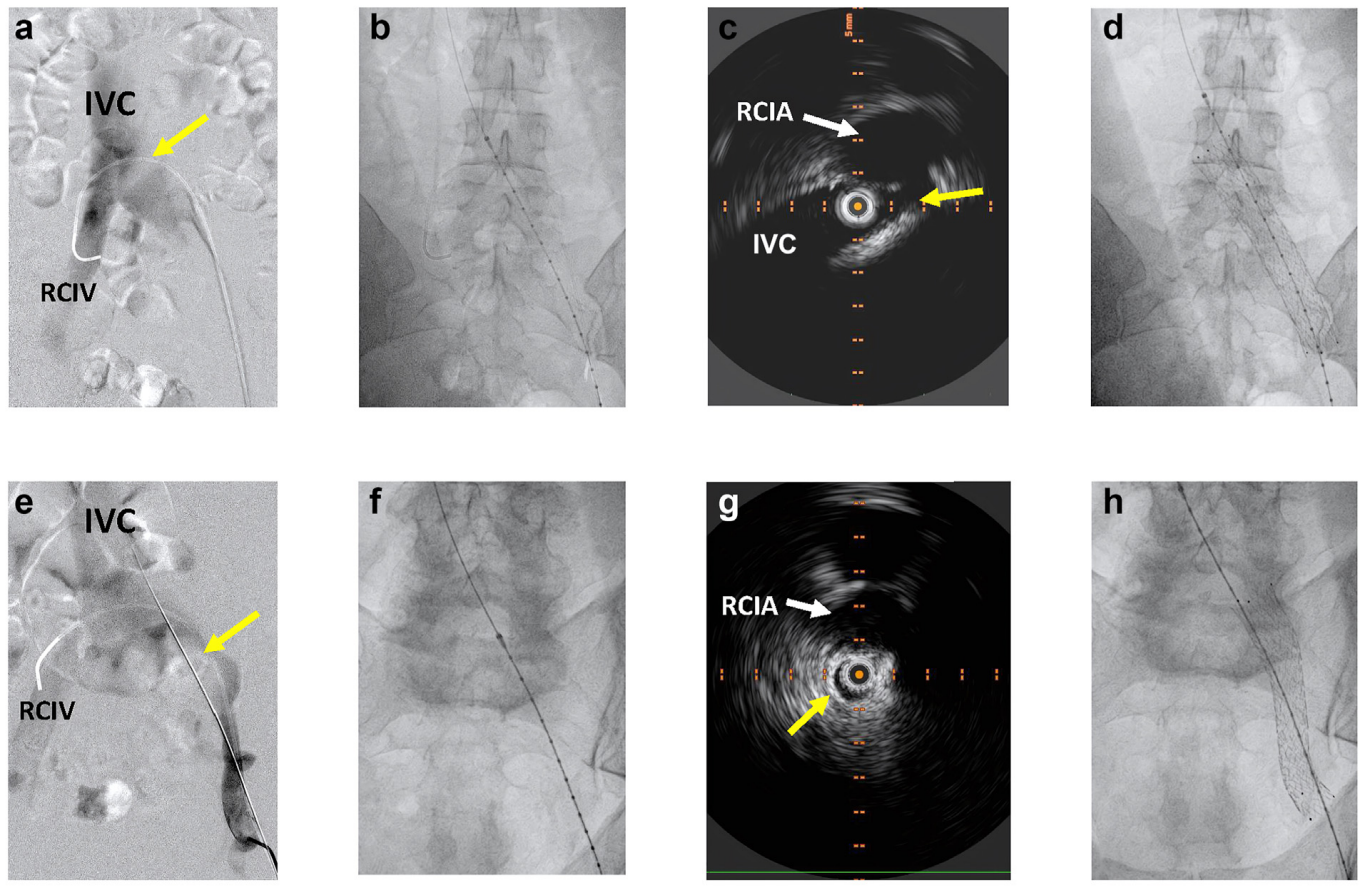
An example showing the process of precise identification of the proximal landing zones at iliocaval confluence. Case 1: **(A)** A cross-over phlebography showing iliocaval confluence. Yellow arrow points to the shadow caused by the right common iliac artery (*RCIA*) compressing the left common iliac vein. **(B)** The tip of the intravenous ultrasound (IVUS) catheter positioned in inferior vena cava (*IVC*), descending into the left common iliac vein (*CIV*) to detect the starting initial site of compression by RCIA at the right side of spinous process. (**C**) The simultaneously IVUS image showing IVC and RCIA (*white arrow*) starting to compress the LCIV (*yellow arrow*). **(D)** The final image after stent implantation showing that the proximal landing zone is left to spinous process. Case 2. **(E)** A cross-over phlebography showing iliocaval confluence. Yellow arrow shows the shadow of RCIA compressing the left CIV. **(F)** The tip of the IVUS catheter is placed in left CIV to detect the starting point of compression by RCIA at the left side of spinous process. **(G)** The simultaneously IVUS image showing the RCIA (*white arrow*) starting compress the left CIV (*yellow arrow*). **(H)** Final image after stent implantation showing that the proximal landing zone situated on the right side of spinous process.

The proximal landing zone is the healthy area proximal to the venous obstruction. Accurate identification of this zone is crucial to ensure sufficient treatment of outflow obstruction. In patients with iliofemoral PTS, the proximal landing zone is typically in the iliocaval confluence. As such, some degree of jailing of the contralateral iliac vein is often unavoidable. To maximize the precision of stent implantation during the procedure, it is highly advisable to use IVUS imaging combined with simultaneous fluoroscopy to exactly define the suitable starting point of the stent.^85^ Owing to anatomical variation of the vessel locations in iliocaval confluence and aortoiliac bifurcation, using just bony landmarks to predict the point of maximum compression can cause missing the compression point or unnecessary extension of stent into the IVC, causing excessive jailing of contralateral iliac vein (Fig 2). If IVUS examination is not accessible, the iliocaval confluence should be determine by phlebography in cross-over technique. In this method, the right iliac vein is catheterized from the left side. Then, phlebography is performed over the catheter starting from the right iliac vein. The operator should pull back the catheter into the left iliac vein upon injecting the contrast. This maneuver visualizes the iliocaval confluence sufficiently (Fig 2). Combining it with preoperative magnetic resonance venography or computed tomography venography is sufficient to properly find the proximal landing point.

## Defining the distal landing zone

The distal landing zone in venous stenting is a critical factor in the success of endovascular treatment for iliofemoral PTS. This area serves as the endpoint for stent placement, ensuring that the stent is anchored in a healthy vein segment (in CVO types 1, 2, and 3) or with adequate inflow (CVO type 4) (Fig 1). The goal is to restore optimal venous return, alleviate symptoms, and prevent re-thrombosis.

**Fig 3:**
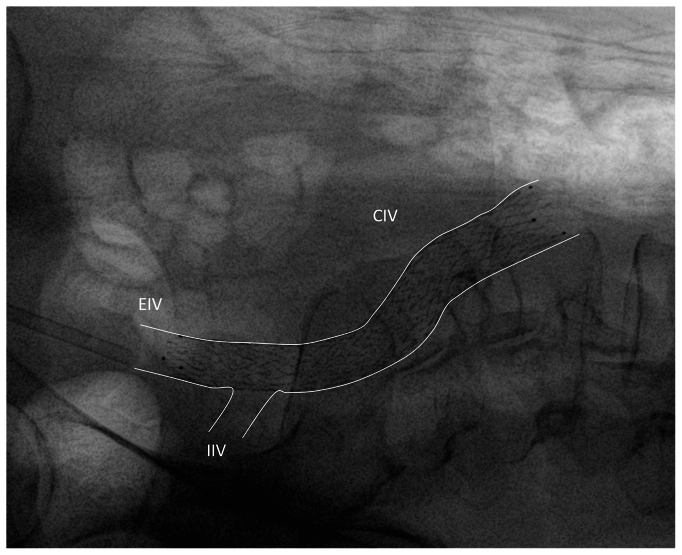
Lateral view of an implanted stent deployment in iliac segment. The external iliac vein (*EIV*) has been selected as the distal landing zone to ensure sufficient anchoring of the stent and reduce the risk of migration. *CIV*, common iliac vein; *IIV*, internal iliac vein.

In patients with iliac PTS (type 2), the distal landing zone is a healthy vein segment, which does not need to be marked precisely before stent deployment. However, the correct choice of stent length and distal landing zone is crucial to prevent stent migration. A review of the existing literature on venous stent migration revealed that 82.6% of the migrating stents measured ≤60 mm in length, 93.6% were ≤14 mm in diameter, and 41.6% of migrations occurred within 30 days of stent placement.^86^ These findings align with data from the US Food and Drug Administration's MAUDE database, which recorded a total of 67 cases of iliac vein stent migration between March 2019 and May 2023.^87^ It is important to ensure that the stent extends distally into the external iliac vein for adequate anchoring (Fig 3). It is advisable to use stents >100 mm long for this type of pathology. Additionally, it is appropriate to perform the postdilation first at the distal segment of the stent and then the point of maximum compression to minimize the risk of stent migration. It should also be noted that this anatomical region is known to have the highest degree of curve.^41^ The reason for not to land at the bottom of the pelvic curve with the stent is to respect the anatomical curve of the iliac vein to avoid tenting of the vein.^88^ In patients with iliofemoral PTS, efforts should focus on the quality of inflow to be sufficient to prevent stent thrombosis. This point is particularly crucial in post-thrombotic conditions extending below the inguinal ligament, where compromised inflow from the common FV (CFV), FV, and DFV can lead to significant increase in risk of stent thrombosis.^14^^,^^89^ Extending below the inguinal ligament and into the CFV to cover existing disease is crucial for optimal long-term outcome. In patients with CVO types 3 or 4, the distal landing zone should be chosen just above the ostium of DFV to avoid jailing the FV or DFV.^82^ If disease extends into the FV and/or DFV, then the long-term outcome is questionable. The magnitude of the infrainguinal vessel disease is a major determinant of stent patency.

Choosing the right size for a stent in patients with chronic iliofemoral venous obstruction is a controversial subject. Accurate sizing is critical to match the inflow and outflow rates,^90^ avoiding to undersize and reduce the risk of migration or oversize which may be one reason of chronic postinterventional back pain,^51^ yet this process is complicated by variable patient anatomy and the altered physiology of post-thrombotic veins. Despite these complexities, there is a range of stent sizes that are most commonly used: 20 to 24 mm for the IVC, 14 to 16 mm for the iliac veins, and 12 to 14 mm when extending into the CFV.^11^ Hence, the decision remains tailored to the individual patient's venous anatomy and the specifics of the obstruction. However, owing to lack of evidence no particular strategy can be recommended.

**Fig 4:**
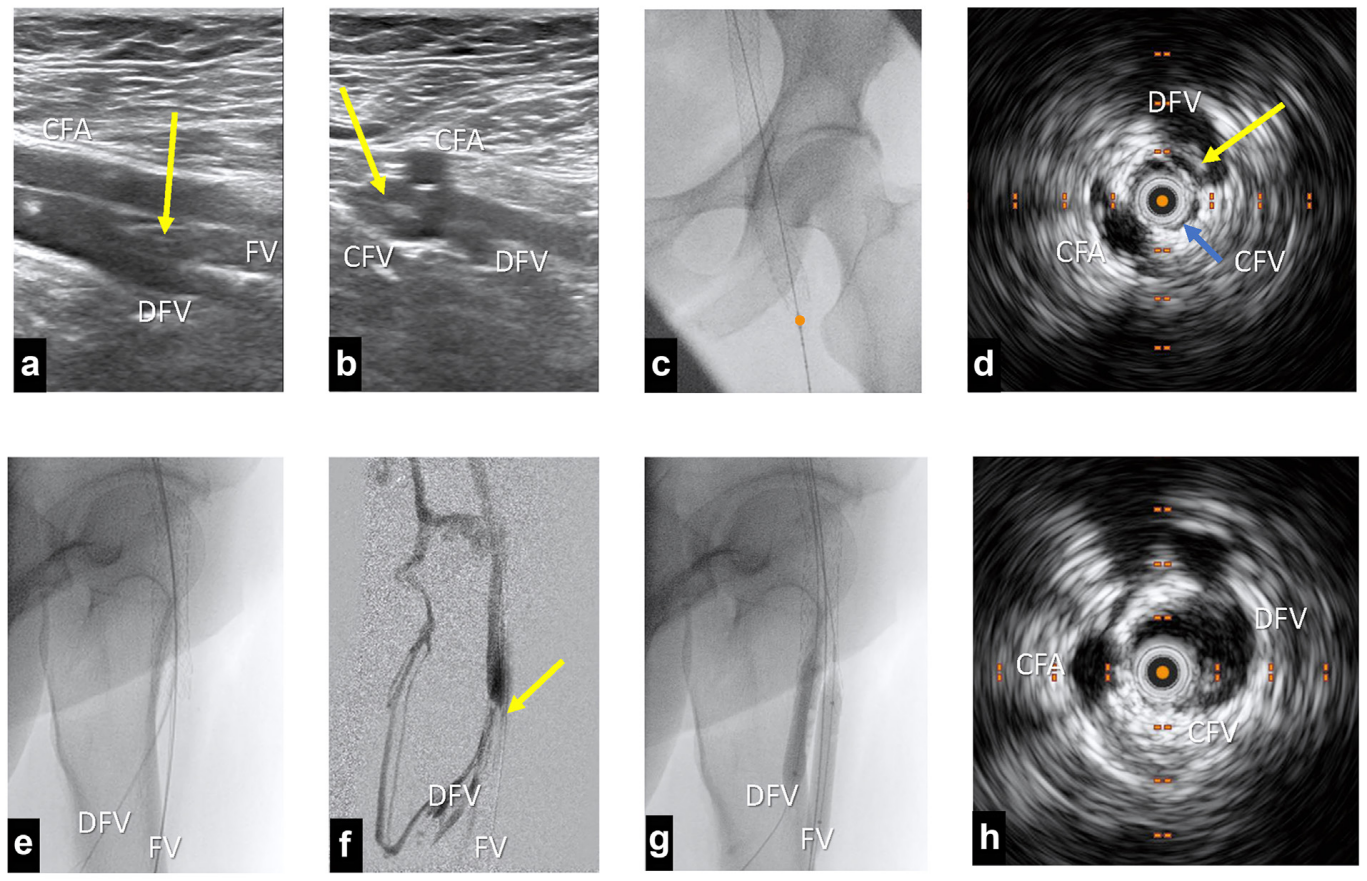
An example showing the role of preoperative DUS and intraoperative IVUS in checking the extension of post-thrombotic changes and how to ensure the single inflow vein can drain the blood into the stents. **(A)** Longitudinal view of the preoperative DUS showing the post-thrombotic involvement (*yellow arrow*) of CFV and FV which leaves the DFV as the single inflow vein. **(B)** Cross-sectional view of the preoperative DUS in same case ensures open and free DFV. Yellow arrow shows the post-thrombotic occluded CFV. **(C)** AP intraoperative fluoroscopy after stent implantation with simultaneous IVUS. The tip of the IVUS catheter (*orange dot*) at distal landing zone checking the DFV. **(D)** Acquired image of IVUS in the same position as **(C)** showing that the post-thrombotic synechiae (*yellow arrow*) blocks the orifice of DFV and hinders the inflow. **(E)** Lateral fluoroscopy image shows the (transjugular) retrograde cannulation of DFV. **(F)** Phlebography shows the post-thrombotic synechiae (*yellow arrow*) blocks the flow into the stents with dominated flow into the collaterals. **(G)** Simultaneous ballooning the FV (antegrade) and DFV (retrograde) to rupture the synechiae. **(H)** IVUS image after ballooning the DF and DFV showing the free pass way from DFV into CFV. *AP*, anteroposterior; *CFV*, common femoral vein; *DFV*, deep femoral vein; *DUS*, duplex ultrasound; *FV*, femoral vein; *IVUS*, intravascular ultrasound.

Although it has been suggested to categorize the inflow as good, fair, or poor, no standardized and universally accepted method exists currently to quantify venous inflow. This uncertainty necessitates a more clinical approach to patient assessment and treatment planning to mitigate the risk of stent thrombosis and optimize long-term patency.^81^ It is reported that the worst outcomes after such interventions are expected in patients whom both of the inflow veins (FV and DFV) are occluded.^25^^,^^91^^,^^92^ As the result, it is imperative to thoroughly assess these inflow vessels preoperatively. In situations where only one of these veins remains patent, ensuring unobstructed blood flow into the stented segments becomes a critical aspect of the intervention. The presence of trabeculations or synechiae over the orifice of the patent inflow vein can disrupt flow dynamics, creating areas of stasis or turbulence that could precipitate thrombosis within the stent. Therefore, careful imaging and, if necessary, intraprocedural management such as balloon angioplasty or even endophlebectomy may be warranted to optimize inflow^93^, ^94^, ^95^, ^96^, ^97^ (Fig 4). Particularly when the obstruction affects the femoral confluence, endophlebectomy plays a crucial role. This surgical procedure involves the removal of intraluminal synechiae and septae from the CFV and its tributaries to restore adequate inflow into the recanalized venous segment. Endophlebectomy helps to establish a clean landing zone and ensures uninterrupted flow from the DFV and other tributaries of CFV into the stented iliofemoral veins.

## Special considerations in reconstruction of iliocaval confluence

Several stent deployment techniques have been developed to address the specific challenges associated with iliocaval confluence reconstruction. These techniques include. The double-barrel technique, skip stent technique, Inverted Y technique, apposition technique, and endophlebectomy.

### Double-barrel technique

**Fig 5:**
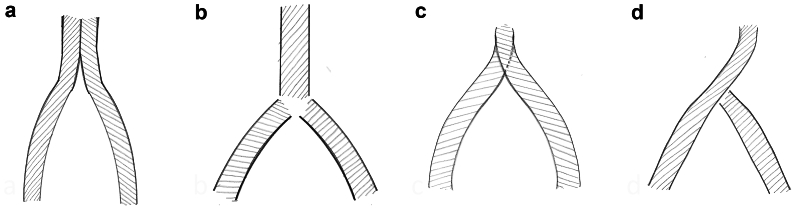
Approaches to reconstructing the iliocaval confluence. (**A**) The double-barrel technique involves placing two parallel stents side by side within the bifurcation, resembling the barrels of a shotgun. **(B)** The skip stent technique is a strategic placement of stents that involves positioning two self-expanding nitinol stents <1 cm apart from an existing caval stent and deploying them at the same time. **(C)** The inverted-Y fenestration technique involves creating a Y-shaped fenestration through one stent to accommodate the unique anatomical configuration of the bifurcation. **(D)** The apposition technique places stents in close contact with each other to ensure a secure and precise fit at the bifurcation site.

The double-barrel technique involves the side-by-side placement of two stents in a parallel configuration across the iliocaval confluence. This approach provides excellent support and coverage of the affected area and is considered the preferred method when feasible owing to its high success rate and lower reintervention rate^98^^,^^99^ (Fig 5, *A*). Currently, there is a lack of evidence from comparative studies on the performance of various dedicated venous stents in a double-barrel configuration, which limits our ability to provide recommendations on stent selection based on design, such as closed cell vs open cell, in these scenarios. Further research is needed to determine the optimal stent characteristics that ensure the best clinical outcomes in the treatment of iliocaval confluence using double-barrel technique.

### Skip stent technique

In this method, a large size venous stent is placed in the IVC and a nitinol stent in each common iliac vein close to the caval stent, leaving a small gap (a skip area) at the level of the iliocaval confluence. This technique aims to provide optimal support to all three venous axes of the confluence without jailing the contralateral iliac stent or creating dead space. It is suggested that this technique does not lead to lower patency rates compared with other techniques^100^ (Fig 5, *B*). This approach allows for a simple stent implantation technique that avoids the creation of a dead space between iliac and caval stents that could disrupt optimal venous flow and lead to thrombotic events. Moreover, there is a decrease in the amount of stent material at the confluence level. This minimization may decrease the risks associated with excessive foreign material, such as induction of neointimal hyperplasia, while still ensuring the necessary structural support for patency.

### Inverted Y technique

The inverted Y technique involves creating a fenestra or window through the side braiding of a stent previously placed across the iliocaval confluence. A second stent is then deployed through this fenestra, forming an inverted Y configuration. This technique is particularly useful in cases of delayed contralateral stenting. However, it has been associated with a higher reintervention rate and lower primary patency, especially in cases of post-thrombotic occlusion^98^^,^^101^ (Fig 5, *C*).

### Apposition technique

The apposition technique involves placing a stent as close as possible to a previously deployed stent across the iliocaval confluence, leaving a small unsupported area between the stents. This technique aims to provide additional support to the affected area. However, it has been associated with a higher reintervention rate owing to restenosis of the unsupported segment. As such, this method has is recommended as the bail-out solution in cases where other methods fail^98^ (Fig 5, *D*).

### Endophlebectomy

Endophlebectomy of the CFV is a surgical procedure that involves the removal of intraluminal synechiae and post-thrombotic trabeculations from the CFV.^93^^,^^94^^,^^97^ This procedure is typically performed in conjunction with iliac vein stenting to restore venous flow in patients with chronic iliofemoral venous occlusion.^96^ The main aim of endophlebectomy is to mitigate the problem of intraluminal synechiae being pushed against the orifices of inflow vessels, which can decrease stent inflow when lesions at the origin of the FV or DFV is stented.^93^ The procedure may be accompanied by arteriovenous fistula (AVF) creation, which can help against the temporary increase in thrombogenicity that can occur with endophlebectomy.^94^ Historically, the placement of the AVF was performed at the caudal end of the endophlebectomy area. However, owing to patency-related problems potentially associated with intimal hyperplasia, a shift toward placing the AVF cranially as a loop shape has been suggested.^102^^,^^103^

An alternative to surgical endophlebectomy is to treat the patients entirely in an endovascular manner. This approach involves recanalization of the occluded venous segment using balloon angioplasty and stent implantation down to the CFV. However, care should be taken to ensure no septae blocks the inflow from inflow veins (FV or DFV) into the stents. This can be assured using IVUS imaging before the stenting to check the distal landing zone at the level of CFV. Additionally, it is highly recommended to check the final result using IVUS imaging after treating patients with this extensive pathology (Fig 4).

The findings of a study comparing endovascular treatment (n = 49 limbs) with hybrid procedure (n = 59 limbs) showed similar cumulative primary patency rates (endovascular, 33.7% vs hybrid, 36.3%) at the 36-month interval. However, endovascular strategy offers several distinct advantages. Specifically, it has a shorter procedure duration, which could mean less anesthesia and potentially a lower risk for the patient. It also results in a shorter hospital stay, which can be beneficial both in terms of patient comfort and decreasing health care costs. Furthermore, the endovascular approach demonstrated fewer postoperative complications and wound healing disorders, leading to better clinical improvement in patients.^82^ However, the choice of treatment should be based on the specific case and the surgeon's experience.^11^^,^^95^

## Postoperative care

Effective postoperative management is a critical component of care for patients who have undergone venous stenting. Follow-up visits typically involve clinical evaluation, imaging studies, and patient-reported outcome measures.^14^^,^^38^^,^^54^ Generally, the first follow-up is recommended within the first month after the procedure, followed by subsequent visits at 3, 6, and 12 months, and annually thereafter.^10^^,^^11^ These time frames facilitate the early identification of any immediate postoperative issues and the monitoring of stent patency over time.

Oral anticoagulation is generally recommended for patients after stent implantation to prevent in-stent stenosis or stent occlusion.^104^^,^^105^ The choice of agent and the duration of therapy should be individualized. Treatment may range from short term (3-6 months for NIVLs)^106^ to long term, depending on the patient's risk factors, the complexity of the reconstruction, and the presence of any stent-related issues.^105^^,^^107^^,^^108^ It is essential to strike a balance between the prevention of complications and the risk of bleeding associated with antithrombotic therapy. The role of antiplatelet therapy is less certain; in our practice, we typically opt for monotherapy, considering the lack of evidence for dual antiplatelet therapy and the heightened bleeding risk.^105^ Close collaboration with a hematologist is recommended to tailor antithrombotic treatment strategies on an individual basis.

In conjunction with pharmacotherapy, mechanical adjunctive measures such as intermittent pneumatic compression devices can be used perioperatively and afterwards to decrease thrombotic risks. Intermittent pneumatic compression increases venous peak velocity, time-averaged maximum velocity, and volume flow^109^ and enables emptying deep veins of the lower limb and preventing stasis.^110^

## Conclusions

Interventional treatment for PTS have greatly improved patient care. The progress in venous stenting emphasizes the importance of careful patient selection, the critical role of inflow for stent success, and the precise identification of landing zones, aided by IVUS examination for enhanced accuracy. The anatomical classification of iliofemoral CVO has potential to serves as a valuable tool for refining the process of selecting suitable candidates for interventional procedures and for providing patients with a comprehensive understanding of their treatment.

## Author Contributions

Conception and design: MB, HJ

Analysis and interpretation: MB, EA, DB, SD, ML, HJ

Data collection: MB, EA, HJ

Writing the article: MB, HJ

Critical revision of the article: MB, EA, DB, SD, ML, HJ

Final approval of the article: MB, EA, DB, SD, ML, HJ

Statistical analysis: Not applicable

Obtained funding: Not applicable

Overall responsibility: MB

## Disclosures

None.
